# Nitrogen-doped reduced graphene oxide for high efficient adsorption of methylene blue

**DOI:** 10.3389/fchem.2024.1484610

**Published:** 2025-01-06

**Authors:** Maoping Kang, Yongli Pei, Ying Zhang, Lihong Su, Yuxiang Li, Hongyu Wang

**Affiliations:** Department of Energy Chemistry and Materials Engineering, Shanxi Institute of Energy, Jinzhong, China

**Keywords:** nitrogen-doped reduced graphene oxide (NRGO), methylene blue (MB), adsorption mechanism, potential application, wastewater treatment

## Abstract

A highly efficient and widely applicable adsorbent for the removal of methylene blue (MB) was created using nitrogen-doped and reduced graphene oxide (NRGO). The effects of NRGO mass, pH, contact time, and the initial MB concentration on the adsorption properties of MB onto NRGO were investigated. The results showed that the adsorption behavior remained stable within the pH range of 2.0–10.0, and the adsorption process gradually reached equilibrium after 24 h. Additionally, the adsorption kinetics and adsorption isotherms were discussed to propose a theoretical adsorption mechanism. Meanwhile, some characterizations including Scanning Electron Microscopy, Energy Disperse X-ray Spectroscopy, X-ray Photoelectron Spectroscopy, X-ray Powder Diffraction, Fourier Transform Infrared Spectroscopy, etc. were used to explore potential adsorption mechanism, which indicated the physisorption caused by π-π bonds was the main adsorption mechanism. NRGO exhibits efficient MB absorption and holds significant potential application for the wastewater treatment.

## Graphical Abstract

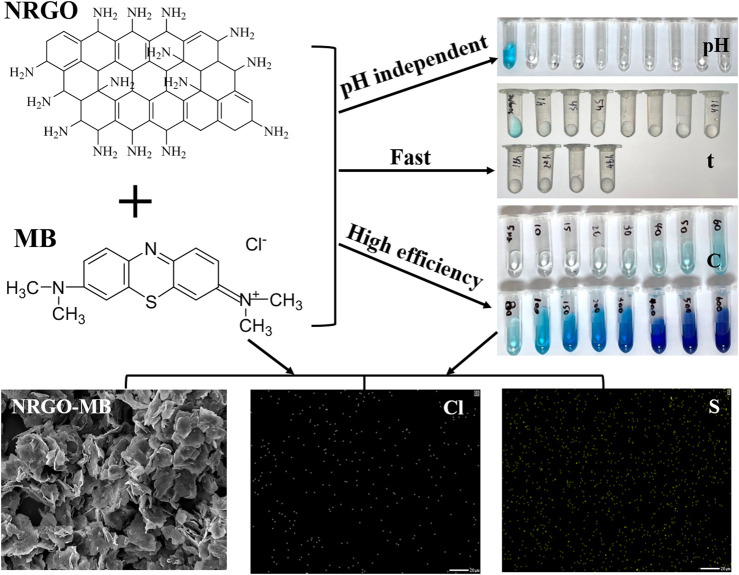

## 1 Introduction

Methylene blue (MB) is a cationic dye extensively utilized in various industries, including textile, food, pulp mill industries (Ang et al., 2022), and the medical field. For instance, in oral medicine, MB can act as a photosensitizer in photodynamic therapy (PDT) for the treatment of oral diseases such as dental caries, periodontitis, and oral mucosal disease via photocatalysis. Nevertheless, the residual MB in wastewater has lots of harmful effects on organisms such as mutagenicity, carcinogenicity and teratogenicity, thereby threatening ecological security and human health because of its high toxicity. Additionally, MB is resistant to natural degradation. If untreated sewage containing MB is discharged into natural water bodies, it will result in severe water pollution, adversely affecting on aquatic organisms and human health (Shitu and Ibrahim, 2014). Consequently, MB has been recognized as one of the major pollutants globally (Fouzia and Nasar, 2020).

To address this issue, various conventional methods such as coagulation, ion exchange, chemical precipitation, electrolysis, reverse osmosis, and electrodialysis have been adopted to remove MB from wastewater. However, these technologies are complicated operation, time-consuming and inadequate to address modern wastewater treatment issues (Dutt et al., 2020). Therefore, there is an urgent requirement for an effective and eco-friendly approach to remove MB to mitigate its harmful impacts. The adsorption-based method has garnered significant attention by researchers due to its high efficiency, rapid action, simple operation, and environmental compatibility. Meanwhile, some adsorbents have been studied as shown in Table 1. Among these adsorbents, graphene-based materials demonstrated a definite advantage because of its large surface area, abundant functional groups (hydroxyl, carboxyl, and epoxide) (Bhat et al., 2022; Gordon-Nuez et al., 2019), low cost, simple preparation and easy modification, etc. However, GO is easy to aggregate to reduce the active site, which will affect its adsorption properties. Also, the separation of nanosized GO from the sewage after adsorption is challenging leading to the waste of adsorbent (Li et al., 2013). Last but not least, adsorbents applicable over a wide pH range are very necessary due to the different pH levels of different water bodies. Therefore, developing the adsorbent with not easily agglomerate, reduced secondary pollution and wide applicability is extremely crucial for the removal of MB.

**TABLE 1:** Adsorption behavior of MB with different reported absorbents.

Adsorbents	Dye concentration (mg/L)	pH	Adsorption efficiency (%)	References
A/C-GO	100	9	94.70	Hridoy Roy and Md Mahmud Kamal Bhuiyan (2024)
ZnFe_2_O_4_/GO (1:5)	10	8	97.37	Aya et al. (2024)
GO	20	6	60.2	Al et al. (2022)
Perlite@GO	500	8	99.7	Iuliano et al. (2024)
γ-AlOOH/3D-rGO	10	7	N/A	Han et al. (2024)
GNS	500	≥8	99	Wang et al. (2024)
Se-ZnFe_2_O_4_/rGO nanohybrids	50	N/A	92.4	Krishnan et al. (2021)

Nitrogen-doped reduced graphene oxide (NRGO) is a new type of carbon nanomaterial synthesized from GO with nitrogen as a doping element, which has the advantage of a large specific surface area due to lamellar overlap (Wu et al., 2020). Meanwhile, the water solubility of NRGO is significantly reduced compared to GO due to the decrease of oxygen-containing functional groups, which indicates that NRGO can hardly dissolve in water resulting in simple removal. In addition, NRGO exhibits non-cytotoxicity up to 100 μg/mL (Liu et al., 2016), which is less cytotoxic than GO (Fu et al., 2016). With the research progress on the unique performance and advantages of NRGO, its application value has been gradually developed. The incorporation of nitrogenous functional groups narrows the band gap of NRGO, reducing the aggregation of GO and enhancing charge transport, conductivity, and charge separation (Bhirud et al., 2015). Thus, NRGO has been used as base material to obtain some photocatalytic composites (Appavu et al., 2016; Meng et al., 2013). The nitrogen doping and ample functional groups improved the photocatalytic activity, enabling NRGO to act as a catalyst to promote the removal of organic pollutants in water (Wei et al., 2021; Zeng et al., 2016). Furthermore, NRGO’s robust π-π and hydrophobic interactions allow it to adsorb phenol and p-nitrophenol, and be reused, indicating its potential as an effective and recyclable adsorbent for removing phenolic chemicals from wastewater (Zhao et al., 2020). Therefore, NRGO is likely to be an alternative adsorbent.

In this study, NRGO was synthesized and utilized as an adsorbent for MB. The effects of different adsorption conditions including NRGO mass, pH, contact time, and initial MB concentration on MB adsorption were comprehensively investigated. Additionally, the adsorption kinetics and isotherms were also explored to elucidate the potential adsorption mechanism. Furthermore, essential characterizations were performed on both NRGO samples before and after MB adsorption to validate the proposed adsorption mechanism. Collectively, this study may provide a highly efficient and widely applicable adsorbent with potential applications in wastewater treatment.

## 2 Results and discussion

### 2.1 Characterizations of NRGO

**FIGURE 1:**
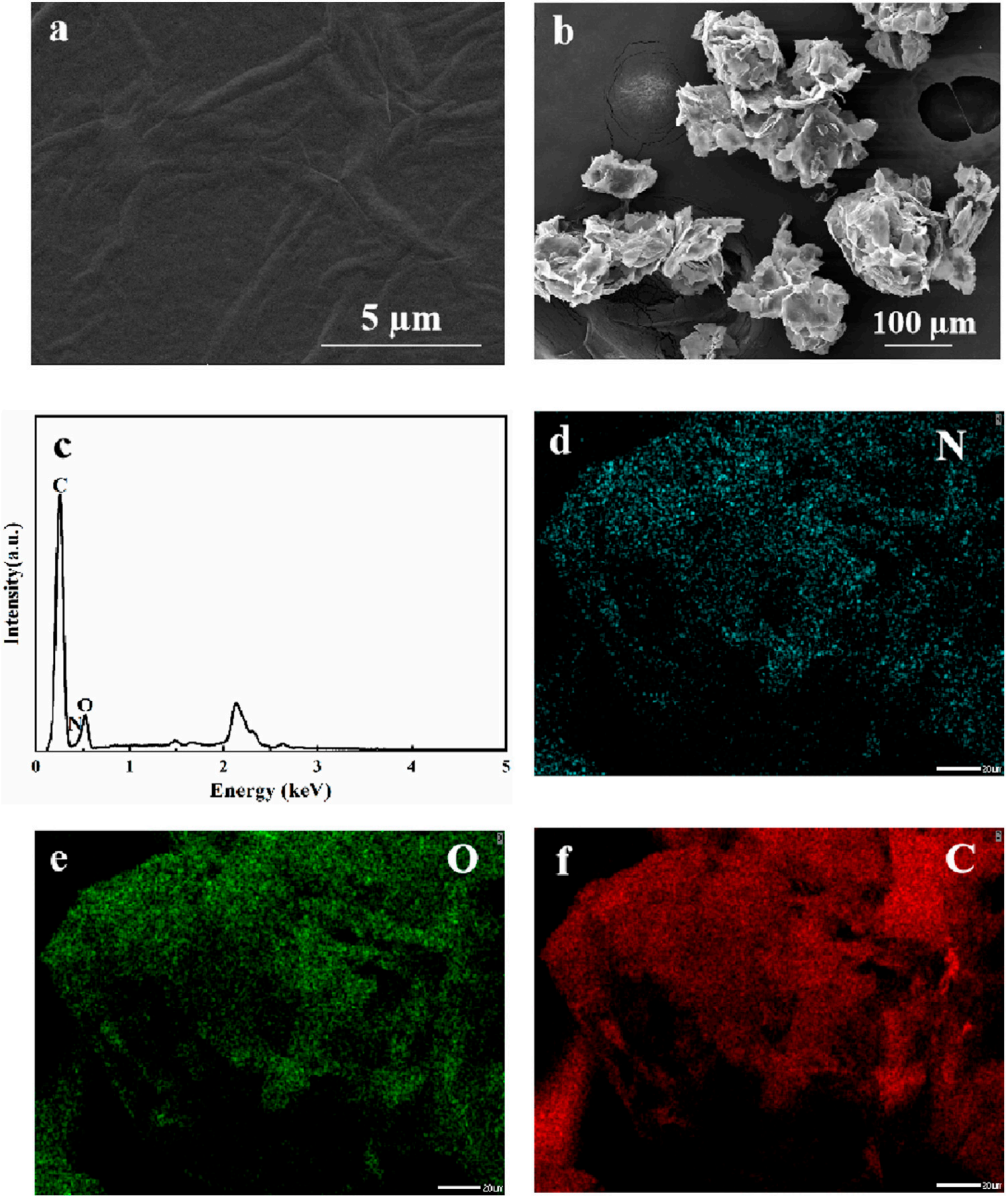
SEM images of GO **(A)** and NRGO **(B)**; The EDX spectrum and elemental mapping images of NRGO **(C–F)**.

NRGO was synthesized through a simple two-step reaction, including the doping of nitrogen and the reduction of graphene oxide (GO). The morphologies of NRGO and GO were observed by Scanning Electron Microscopy (SEM). The results showed that the microstructure of NRGO (Figure 1B) exhibited a loose, multifold, three-dimensional stacked layer with wrinkles and pores while that of GO (Figure 1A) was a relatively flat layer with some wrinkles, which is consistent with the previous study (Gordon-Nuez et al., 2019). Meanwhile, the Energy Disperse X-ray spectroscopy (EDX) spectrum ((Figure 1C and elemental mapping images (Figures 1D–F) confirmed that nitrogen was clearly observed and uniformly distributed in the synthesized NRGO, distinguishing it from GO, which is composed solely of carbon and oxygen. This observation confirms that NRGO was successfully synthesized by through nitrogen doping.

**FIGURE 2:**
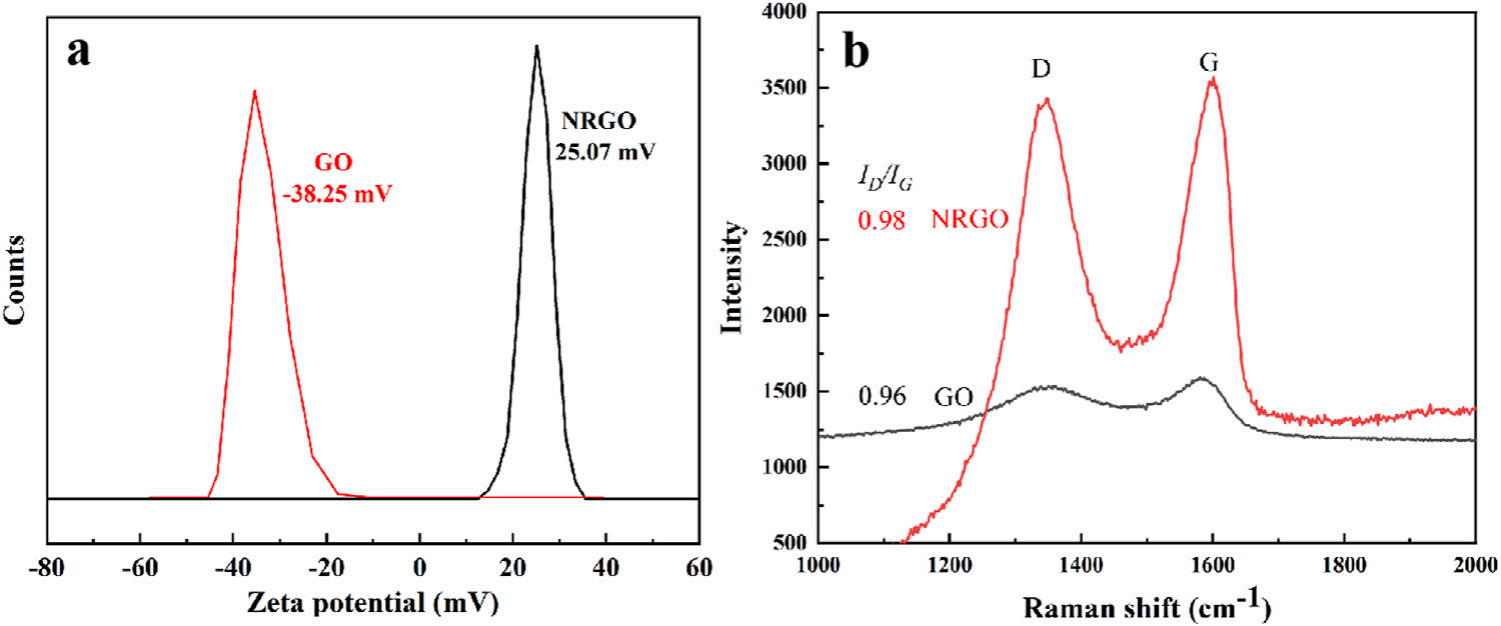
Zeta potential **(A)** and Raman spectra **(B)**.

Surface charge and Raman were used to further verify the reduction and functionalization of GO. As shown in Figure 2A, GO showed the negative potential (−38.25 mV), while NRGO showed the positive potential (25.07 mV), which possibly indicated graphene oxide was aminated. Meanwhile, the change of carbon frame structure was characterized by Raman spectrum. As shown in Figure 2B, the D and G band were clearly observed for GO and NRGO at 1,349 cm^−1^, 1587 cm^−1^, and 1349 cm^−1^, 1601 cm^−1^, respectively. The G band of NRGO was slightly split and had a slight peak shift, which indicated GO was reduced and functionalized (Song et al., 2012). Also, the *I*_*D*_ */I*_*G*_ of NRGO was higher than that of GO, which indicated the amino groups were modified to the edge or surface of the graphene.

**FIGURE 3:**
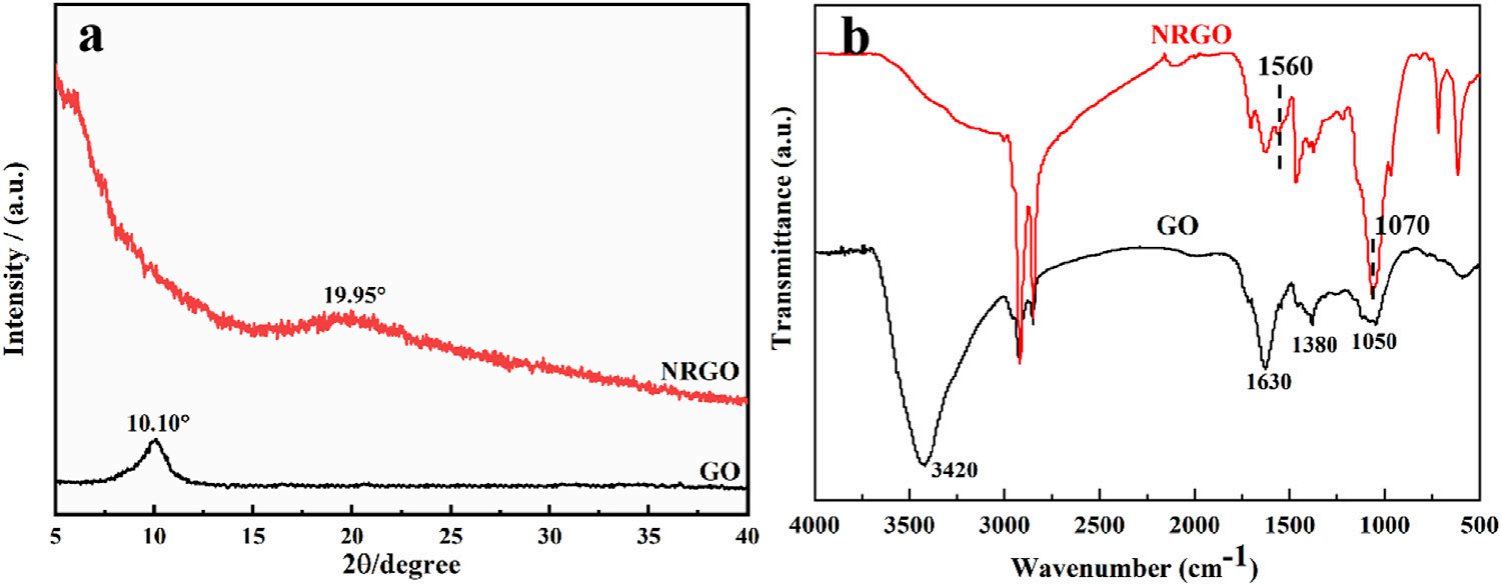
XRD **(A)** patterns and FTIR spectra **(B)** of GO and NRGO.

Moreover, X-ray powder diffraction (XRD) and Fourier transform infrared spectroscopy (FTIR) were used to characterize the changes in structure and functional groups before and after the modification of GO. As shown in Figure 3A, the XRD patterns exhibited an intense characteristic diffraction peak at 2θ = 10.10° for GO and a weak characteristic peak at 2θ = 19.95° for NRGO, attributed to the reduction of oxygen-containing functional groups of GO, leading to a significant decrease in interlayer spacing (Zheng et al., 2013; He et al., 2013). Furthermore, FTIR was used to characterize the changes in functional groups of GO and NRGO. As shown in Figure 3B, the FTIR spectrum of GO displayed a broad and intense peak at 3,420 cm^−1^, corresponding to -OH stretching vibration peak. Additionally, the other four adsorption peaks of GO are displayed at 1720 cm^−1^, 1,630 cm^−1^, 1,380 cm^−1^ and1050 cm^−1^, indicative of the stretching vibration absorption peak of C=O in aryl carboxylic acids, the stretching vibration absorption peak of the C=C form six-membered ring skeleton, the deformation vibration absorption peak of O-H in C-OH group, and the stretching vibration absorption peak of C-O, respectively (Liu et al., 2014; Yang et al., 2020).In contrast, the characteristic absorption peaks of oxygen-containing functional groups in NRGO at 3,420 cm^−1^, 1720 cm^−1^, 1,630 cm^−1^, and 1,380 cm^−1^ O were significantly weakened or absent as expected, confirming the reduction of GO. Instead, a new adsorption peak representing a carbon-nitrogen heterocyclic ring appears at 1,070 cm^−1^ (Yuan et al., 2017), and 1,560 cm^−1^ for bending vibration peak of N-H, indicating the successful introduction of N in the graphene lattice. These results suggested that the oxygen-containing groups in GO were decreased, and the N were doped during the synthesis process.

**FIGURE 4:**
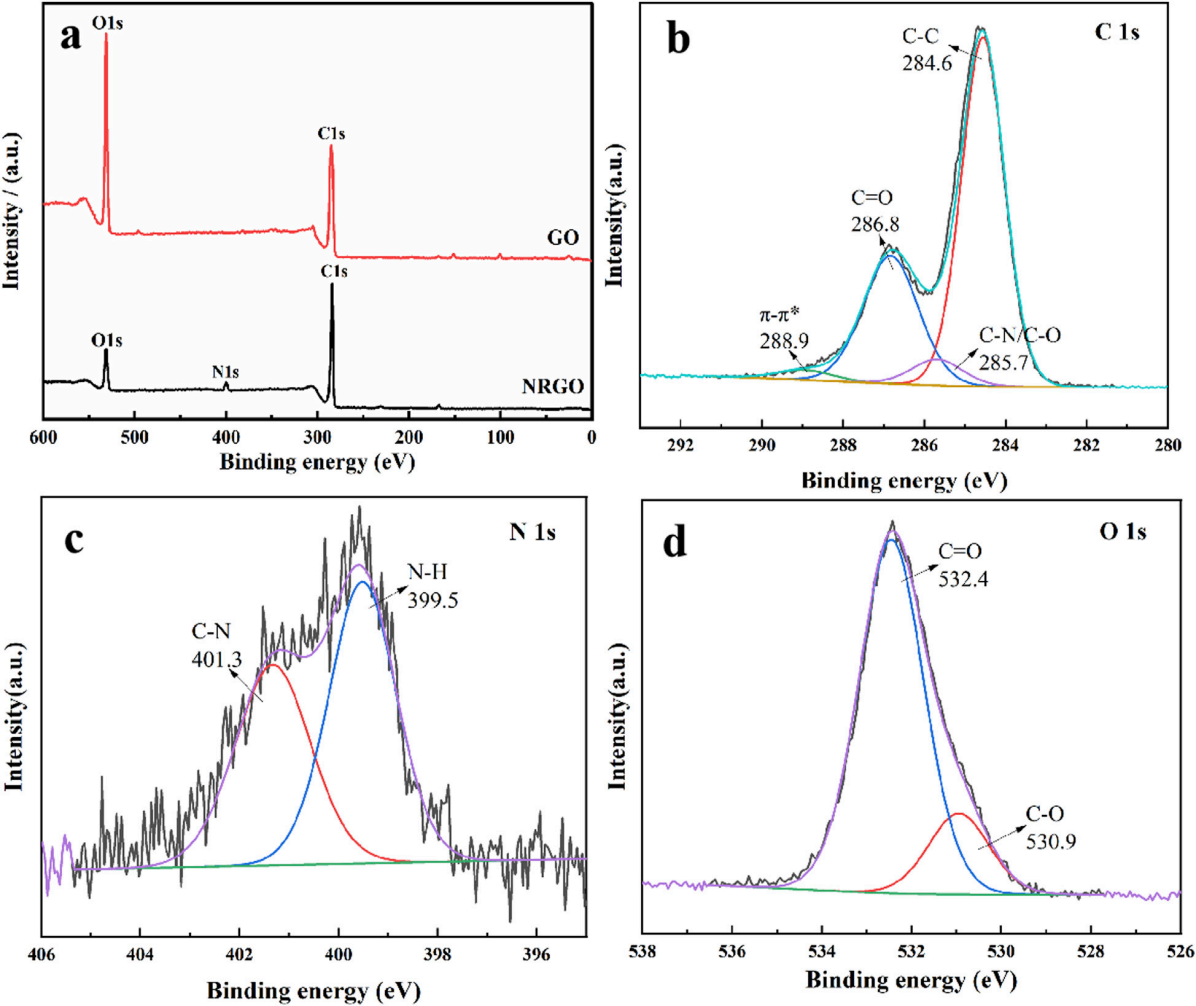
XPS survey spectra of GO and NRGO **(A)**. **(B)** C 1s **(B)**, N 1s **(C)**, and O 1s **(D)** spectra of NRGO.

Additionally, X-ray photoelectron spectroscopy (XPS) was utilized to verify the elemental composition, and the valence bond form of NRGO. As shown in Figure 4A, the N element was clearly observed after N-doping on GO, whereas GO only contained O and C, indicating successfully doped of N element. Additionally, the C:O ratio in NRGO increased significantly compared to GO, which indicated GO was also reduced in obtaining N-doped reduced graphene oxide. Also, C1s (Figure 4B), N1s (Figure 4C), and O1s (Figure 4D) spectra were studied to analyze the possible valence bond. As shown in C 1s spectrum (Figure 4B), the four characteristic peaks were observed, including C-C (284.6 eV), C-N/C-O (285.7 eV), C=O (286.8 eV), and π-π^*^ (288.9 eV). The higher peak area of C-C indicated the reduction of GO. The appearance of C-N indicates that the amino group in oleoamine may be linked to GO. In Figure 4C, the split peaks of N 1s were observed as C-N (401.3 eV) and N-H (399.5 eV), which indicated the GO was aminated. These results indicated GO was reduced and functionalized by oleylamine through the formation of C-N and N-H, which was also verified by the FTIR results.

### 2.2 Adsorption performance

#### 2.2.1 The adsorption behavior of NRGO and RGO

**FIGURE 5:**
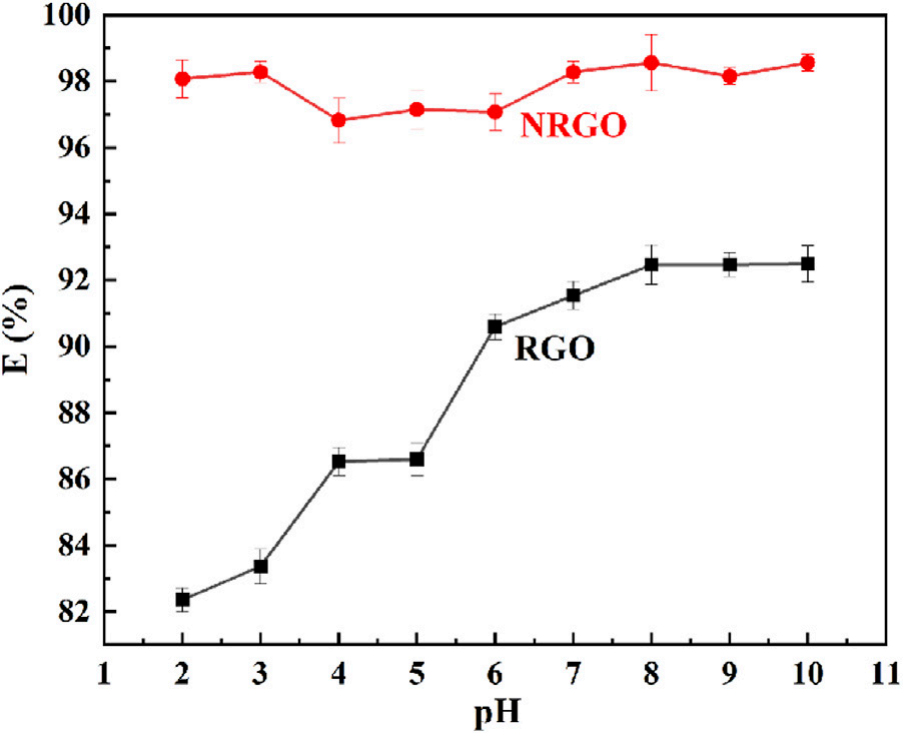
The adsorption efficiency of NRGO and RGO at different pH values.

The obtained RGO was chosen because it was the reduced GO and a non-water-soluble solid, similar to NRGO. 0.03 g of NRGO and RGO was employed to adsorb MB at different pH values. As shown in Figure 5, the adsorption efficiency of GO reached maximum of 92.31% at pH 8, 9 and 10. Nevertheless, the adsorption efficiency was as low as less than 88% under acidic conditions, which indicated that the adsorption behavior of RGO was dependent on the pH values. As for NRGO, the adsorption efficiency for MB remained almost as high as 97% over a wide pH range, which indicated that the adsorption behavior was independent on the pH value of the water body. In summary, NRGO exhibited higher adsorption efficiency for MB than RGO at any pH value, which will be applied in various fields. Therefore, NRGO was chosen as the adsorbent for the further study.

#### 2.2.2 Effect of adsorbent dose

**FIGURE 6:**
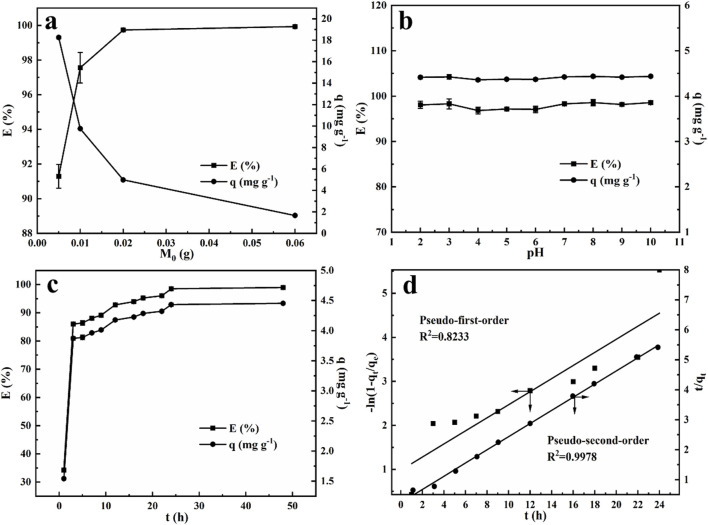
Effect of adsorbent dose **(A)**, initial pH **(B)** and contact time **(C)** on MB adsorbed by NRGO; adsorption kinetics of MB absorbed by NRGO **(D)**.

To explore the effect of the adsorbent dose, varying amounts of NRGO powder (0.005 g, 0.01 g, 0.02 g and 0.06 g) was introduced into 1 mL of 100 mg/L MB solution and shaked for 24 h at room temperature. Figure 6A illustrated the correlation between the initial mass of NRGO and the resulting adsorption performance. The results indicated that the adsorption efficiency increased with the mass of NRGO. Meanwhile, the adsorption efficiency was approximately balanced when the adsorbent dosage reached 0.02 g, suggesting that this amount provides an optimal number of adsorption sites for 1 mL of a 100 mg/L MB solution. Consequently, a dosage of 0.02 g was selected as the optimal adsorbent dosage for following experiments.

#### 2.2.3 Effect of pH for adsorption MB

The pH of the solution significantly influences the adsorption of MB. In this study, MB solutions (30 mg/L) with varying pH values (2.00–10.00) were prepared by adjusting the pH values with NaOH solution (0.1 M) and HCl solution (0.1 M). 0.002 g NRGO powder was added to 300 µL of the above MB solution with different initial pH values, respectively. As shown in Figure 6B, the initial pH of MB solution had a negligible effect on the adsorption efficiency across the acidic to alkaline range. The adsorption efficiency remained consistently around 98% when pH was adjusted to between 2 and 10, indicating that NRGO was a stable and efficient adsorbent in the removal of MB, independent of pH variations. This characteristic positioned NRGO as a potential adsorbent for the actual sample with a wide range of pH. Additionally, the pH of the original MB solution was measured as 6.30. Given the minimal impact of initial pH on the adsorption efficiency, the natural pH of MB solution was utilized for subsequent experiments.

#### 2.2.4 Effect of adsorption time and adsorption kinetics

Investigating the effect of time on adsorption is essential for achieving rapid and efficient adsorption. 0.002 g NRGO was added to MB solution (300 μL, 30 μg/mL), and the mixtures were centrifuged after varying interaction time from 1 h to 48 h. According to Figure 6C both the adsorption efficiency and capacity increased rapidly within 1 h, after which the adsorption rate slowed down with the time increased, indicating that the adsorption sites on NRGO were gradually occupied by MB and approached saturated. Meanwhile, the adsorption efficiency almost stabilized after 24 h, indicating that the adsorption efficiency and capacity kept constant and reached the adsorption equilibrium. Therefore, an adsorption time of 24 h was determined to be optimal for the following experiments.

To explore the adsorption mechanism of the adsorption behavior, the common adsorption kinetic models were investigated using the pseudo-first-order (PFO) and pseudo-second-order (PSO) kinetic models according to the following Equations 1, 2. Therein, the PFO model described a physisorption process where the adsorption process is related to the number of reaction sites and the diffusion rate of the adsorbate, and is expressed in Equation 1 (Haque et al., 2020):

$$-\ln\left(1-\frac{q_{t}}{q_{e}}\right)=k_{1}t$$ (1)

The PSO model reflects the chemisorption process, involving the formation of chemical bonding between adsorbate and adsorbent through electron sharing or exchange, and is expressed in Equation 2 (Haque et al., 2020):

$$\frac{t}{q_{t}}=\frac{1}{k_{2}q_{e}^{2}}+\frac{t}{q_{e}}$$ (2)

Where *q*_*t*_ is the adsorption capacity at time *t* (mg/g), *t* is adsorption time (h), *q*_e_ is equilibrium adsorption capacity of MB (mg/g), *k*_1_ (h^−1^) is the Lagergren rate constant of PFO, *k*_2_ g/(mg·h) is the rate constant of PSO. As shown in Figure 6D, the PSO model, with a correlation coefficient (*R* ^2^) of 0.9978, provided a better fit to the data than the PFO model, with a *R* ^2^ of 0.8233. Moreover, as shown in Table 2, the adsorption capacity *q*_cal_ calculated by the PSO model closely approximated the experimental adsorption equilibrium capacity *q*_exp_. This further proved the applicability of PSO model to the adsorption process, indicating the adsorption rate was controlled by chemisorption mechanism, which involves the electronic sharing and exchange between NRGO and MB.

**TABLE 2:** Kinetics models for the adsorption of CHX on NRGO.

	Pseudo-first-orderln (1−q_t_/q_e_) = −k_1_t	Pseudo-second-order t/q_t_ = 1/(k_2_ q_e_^2^)+t/q_e_		
Model equations	y = 0.9817x+0.1487	y = 0.2715x+0.2678		
Correlation	*R* ^*2*^	*q*_cal_ (mg g^−1^)	*q*_exp_ (mg g^−1^)	*R* ^*2*^
	0.8233	4.61	4.45	0.9978

#### 2.2.5 Effect of the initial concentration of MB and adsorption isotherms

**FIGURE 7:**
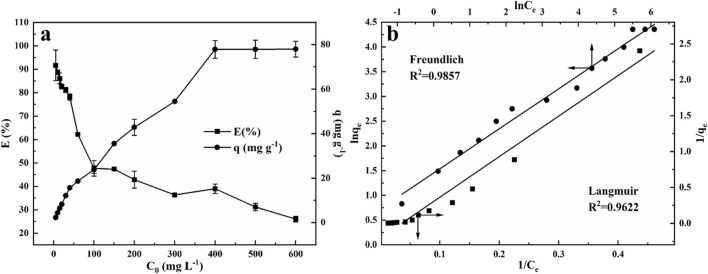
Effect of the initial concentration of MB solution **(A)** and Langmuir and Freundlich adsorption isotherms **(B)** for the adsorption of MB onto NRGO.

To assess the effect of initial MB concentration on the adsorption performance, 0.002 g NRGO powder was added to 1 mL MB solution with varying concentrations ranging from 5 to 600 mg/L. As shown in Figure 7A, the adsorption efficiency decreased while the adsorption capacity increased as the MB concentration increased, which was attributed to the finite number of reactive sites available on NRGO. Notably, as the initial MB concentration increased from 5 mg/L to 100 mg/L, there was a marked decrease in adsorption efficiency and a significant increase in adsorption capacity. Upon further increasing the MB concentration from 100 mg/L to 400 mg/L, the decline in adsorption efficiency and the rise in adsorption capacity continued, but at a decelerating rate. Ultimately, at an initial MB concentration of reached 600 mg/L, the adsorption capacity remained consistent at ∼78 mg/g, indicating that the adsorption sites on NRGO were nearing saturation.

In addition to kinetics studies, the adsorption isotherms were examined to explore the adsorption performance. The experimental data of the effect of the initial MB concentration were fitted with Langmuir and Freundlich isotherm models according to Equations 3, 4.

The Langmuir model, which describes the equilibrium condition of monolayer homogeneous adsorption is expressed as Equation 3 (Wang and Guo, 2020):

$$\frac{1}{q_{e}}=\frac{1}{k_{L}q_{m}c_{e}}+\frac{1}{q_{m}}$$ (3)

The Freundlich model, which represents the multi-layer adsorption on heterogamous surfaces is expressed as Equation 4 (Wang and Guo, 2020):

$$\ln q_{e}=\ln K_{F}+\frac{1}{n}\ln C_{e}$$ (4)

Where *C*_e_ (mg/g) is the equilibrium concentration, *q*_e_ (mg/L) is the adsorption capacity at equilibrium. The Langmuir constants *q*_m_ (mg/L) and *K*_L_ (L/mg) are related to the adsorption capacity and energy, respectively, and were calculated from the intercept and slope of the plot of 1/*q*_e_ versus 1/*C*_e_, respectively. The Freundlich constants *K*_F_ (mg/g) and 1/n are related to the adsorption capacity and intensity, respectively, and were determined from the intercept and slope of the plot of ln *q*_e_ versus ln *C*_e_, respectively.

The plots of the Langmuir and Freundlich isotherm models, along with the data in detail, were displayed in Figure 7B; Table 2, respectively. The results showed that the adsorption performance was both in accordance with Langmuir and Freundlich models, as evidenced by the high correlation coefficients (*R* ^2^) This suggested that both monolayer and heterogeneous adsorption appeared in the adsorption process. According to Table 3, the theoretical maximum adsorption capacity (*q*_m_) was calculated to be 27.90 mg/g according to the Langmuir isotherm. As for the Freundlich constants, *K*_F_ gave an adsorption capacity as 2.949 mg/g, and the value of n was 2.029 (1 < *n* < 10), which indicated the adsorption process of NRGO was obviously heterogeneous (Tanzifi et al., 2018).

**TABLE 3:** Langmuir and Freundlich adsorption isotherm parameters for MB on NRGO.

	Langmuir			Freundlich		
Material	*q*_*m*_ (mg g^−1^)	*K*_*L*_ (L mg^−1^)	*R* ^*2*^	*K*_*F*_ (mg g^−1^)	n	*R* ^*2*^
NRGO	27.90	0.2026	0.9622	2.949	2.029	0.9857

### 2.3 Adsorption mechanism

The possible adsorption mechanism of NRGO for MB can be roughly delineated into three points: 1) The microstructure of NRGO. SEM image in Figure 1B showed that NRGO had more folds and pores, and the three-dimensional lamellar structure of NRGO was conducive to improving the adsorption efficiency of MB. 2) The π-π interaction between NRGO and MB. According to the previous reports, a large number of benzene rings on NRGO have been proved to be effective adsorption of phenols through π-π bonds (Zhao et al., 2020), potentially facilitating interactions with the aromatic groups of MB molecules. 3) The formation of new chemical bonds. The kinetic study results indicated that the adsorption of MB by NRGO was more in accordance with pseudo-second-order kinetics, suggesting that the chemisorption predominantly governs the adsorption rate. Therefore, some necessary characterizations were conducted to further elucidate the adsorption mechanism.

**FIGURE 8:**
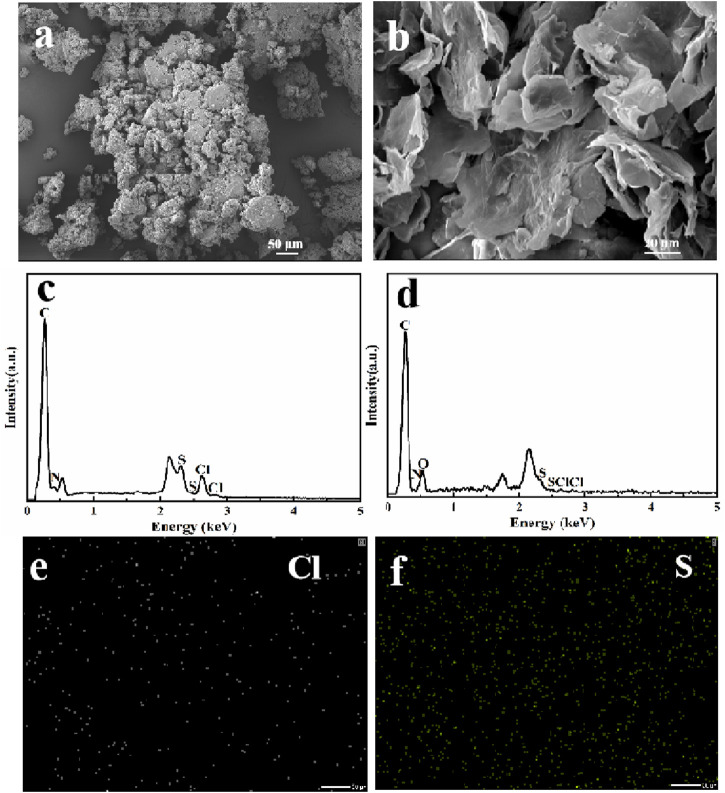
SEM images of MB **(A)** and NRGO-MB **(B)**, and EDX spectra and elemental mapping images of NRGO-MB **(C–F)**.

The intuitive observation of microscopic morphologies could directly reflect the adsorption of MB. Thus, SEM was employed to observe the morphologies change of before and after MB adsorption. As shown in Figure 8A, MB presented a stacked particles form. After the adsorption of MB (Figure 8B), NRGO-MB still kept a stacked lamellar structure, similar to that of NRGO. However, MB was not observed clearly on the surface of NRGO-MB because of the encapsulation of MB by the lamellar structure of NRGO. To further confirm the adsorption of MB by NRGO, EDX analysis was conducted. As shown in Figure 8D, the result showed the presence of carbon, oxygen, nitrogen, chlorine, and sulfur in NRGO-MB, which was consistent with the elements present in MB (Figure 8C). Additionally, the mapping images showed uniformly dispersion of chlorine and sulfur elements on the surface of NRGO-MB (Figures 8E, F). Furthermore, as depicted in the inset image of (Figure 8D), the blue coloration associated with MB appeared lighter or even disappeared after the adsorption by NRGO, indicating successfully adsorption of MB by NRGO.

**FIGURE 9:**
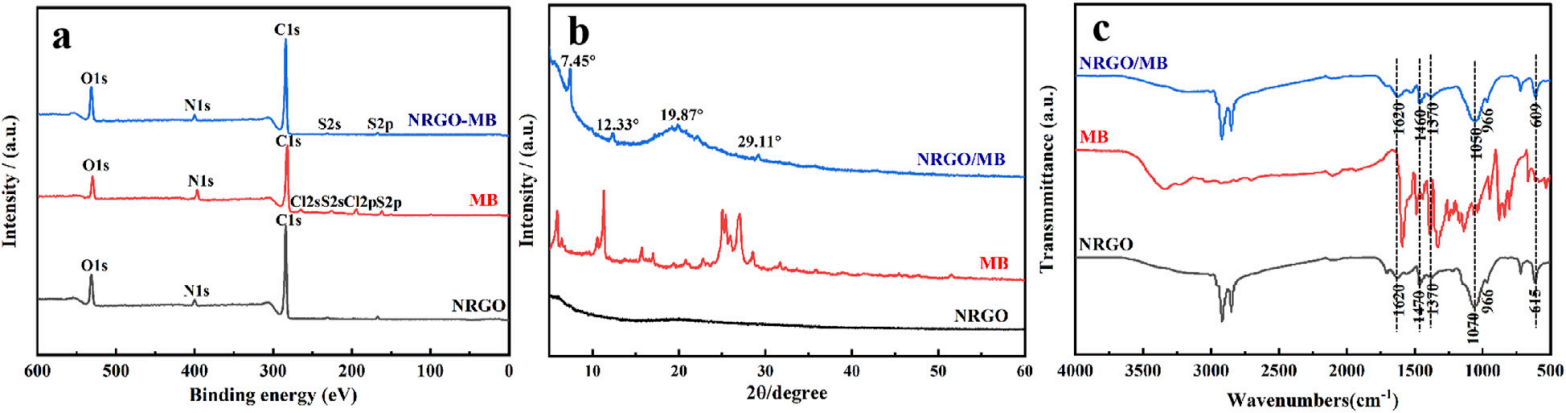
XPS spectra **(A)**, XRD patterns **(B)** and FTIR spectra **(C)** of NRGO, MB and NRGO-MB.

In addition, XPS was utilized to further analyze the elements before and after MB adsorption. As shown in Figure 9A, the NRGO surface was predominantly composed of carbon, oxygen and nitrogen. Apart from carbon, oxygen and nitrogen, the MB surface also contained chlorine and sulfur. Compared with NRGO, sulfur was observed on the surface of NRGO-MB, which aligned with the elemental analysis results of MB, suggesting the successful adsorption of MB onto NRGO. The XRD patterns, as shown in Figure 9B, revealed a shift in the characteristic peak from 2θ = 19.98° for NRGO to 2θ = 19.87° for NRGO-MB, indicating an increased interlayer spacing due to the adsorption of MB on NRGO surface. Moreover, three other characteristic peaks at 2θ = 7.45°, 2θ = 12.33° and 2θ = 29.11° were clearly observed in NRGO-MB, which closely matched with XRD pattern of MB, which indicated that MB was successfully loaded onto NRGO to form NRGO-MB. Furthermore, FTIR was used to characterize the functional groups before and after MB adsorption. As shown in Figure 9C, no significant changes or new peaks were observed in the infrared spectra of NRGO after adsorbing MB, which indicated that the adsorption process was attributed to physisorption through π-π interactions between NRGO and MB.

Taking into account the adsorption kinetic, isotherms, and aforementioned characterizations, the adsorption mechanism was attributed to physisorption attracted by the π-π bonds between NRGO and MB.

## 3 Conclusion

In this study, NRGO was successfully synthesized via amination of GO and subsequently applied for MB adsorption. The effects of NRGO dosage, pH, contact time, and initial MB concentrations on adsorption were thoroughly examined, alongside the adsorption kinetics and isotherms. The results indicated that NRGO exhibited superior and consistent adsorption capacity towards MB at a wide pH range, with the adsorption process following the PSO kinetic model as well as Langmuir and Freundlich isotherm models. Furthermore, the potential adsorption mechanism, attributed to physisorption through π-π interactions between NRGO and MB, was verified through essential characterizations including SEM, EDX, XRD, XPS, and FTIR. These findings highlight the potential application value of NRGO in environmental treatment.

## 4 Experiment section

### 4.1 Materials

Graphene oxide (GO) was prepared in our previous study. The chemicals used in the experiments, including N, N-Dimethylformamide (DMF), 1-(3-Dimethylaminopropyl)-3-ethylcarbodiimide hydrochloride (EDC), cis-9-Octadecenylamine, trichloromethane, petroleum, ethanol, sodium hydroxide (NaOH), hydrochloric acid (HCl) and methylene blue (MB), were all purchased from Chengdu Kelong Chemical Reagent Company (China), and used without further purification. Deionized water (18.25 MΩ cm) was obtained using ULUPURE Water Purification System (Chengdu, China) and was used to prepare all aqueous solutions.

### 4.2 Preparation of NRGO and RGO

NRGO was prepared according to the previous reports. In brief, 0.30 g of GO was weighed and dispersed in 50.0 mL DMF under sonication for 30 min and stirring for 1 h. Then, 0.46 g of EDC was dissolved in 20.0 mL DMF by sonication. The EDC solution was added to GO dispersion, stirred for 2 h and sonicated for 30 min. Subsequently, 0.27 g of NHS was dissolved to 10.0 mL DMF under sonication and then was added to the above reaction system, stirred for 2 h and sonicated for 30 min to ensure complete reaction. Next, 1.28 g of olamine was weighed and added to 10.0 mL of chloroform. After the olamine completely dissolved, the solution was added to the above reaction system. Then the mixture was treated by sonicated at 40°C for 30 min and stirred overnight. The obtained material was filtered through organic membrane (0.22 μm), followed by washing with petroleum ether and ethanol. Finally, the modified graphene oxide was obtained by freeze-drying and marked as N doped reduced graphene oxide (NRGO).

Reduced graphene oxide was prepared as follows:the 0.5 mg/mL GO solution was transferred to polytetrafluoroethylene autoclave at 120°C for 8 h, and the reduced material was obtained as RGO.

### 4.3 Characterization of the samples

The surface morphologies of GO, NRGO, MB, and the composite after MB adsorption were examined by environmental scanning electron microscope (ESEM, JSM-IT500, JEOL Company, Japan). Materials compositions were analyzed by energy disperse X-ray spectroscopy (EDX, JEOL Company, Japan) and X-ray photoelectron spectroscopy (XPS, Kratos, Britain). Changes in the crystal structure of the materials were measured by X-ray powder diffraction (XRD, X’Pert Pro, Philips, Netherlands). The alterations of functional groups of the material were determined using a Fourier transform infrared spectrometer (FTIR, Nicolet IS10, Thermo, America) with KBr pellets in a scanning range of 500–4000 cm^−1^. The change of frame structure was measured by Raman spectrometer (Jobin Yvon S.A.S., HORIBA). The surface charge was detected by zeta potential analyzer (Zetasizer-ZS series, England).

### 4.4 Batch adsorption experiment

Batch adsorption tests were performed to assess the impact of various factors on the adsorption property of MB onto NRGO. The prepared NRGO was grinded into powder and the solid MB powder was dissolved in deionized water to prepare a methylene blue solution for subsequent experiments. For the adsorption experiments, different doses of NRGO with 300 µL of 0.3 mg/mL of MB solution were firstly optimized to ensure the adsorption efficiency as high as possible. Subsequently, a certain amount of NRGO was introduced into MB solution of varying initial concentrations (to study the effect of concentration) at various pH values (for pH experiment) for different contact time (forvadsorption kinetic experiment). After adsorption at room temperature (25°C), the absorbance of their supernatant was measured by U-2910 UV–Vis spectrophotometer. The final concentration of MB after adsorption was calculated to determine the adsorption rate (*E*,%) and adsorption capacity (*q*, mg/g) according to experimental Equations 5, 6 as follows:

$$E=\left(C_{0}-C\right)/C_{0}\times 100\%$$ (5)

$$q=\left(C_{0}-C\right)\times V/M$$ (6)

Where *E* is the adsorption efficiency of MB (%), *q* is the adsorption capacity of MB (mg/g), *C*_0_ and *C* are the initial and final concentration of MB solution (µg/mL), respectively, *V* is the volume of MB solution (L), *M* is the mass of the adsorbent (g).

## Data Availability

The original contributions presented in the study are included in the article/supplementary material, further inquiries can be directed to the corresponding author.
